# Contactless integrated photonic probes: fundamentals, characteristics, and applications

**DOI:** 10.1007/s12200-024-00127-1

**Published:** 2024-08-05

**Authors:** Guangze Wu, Yuanjian Wan, Zhao Wang, Xiaolong Hu, Jinwei Zeng, Yu Zhang, Jian Wang

**Affiliations:** 1 Wuhan National Laboratory for Optoelectronics and School of Optical and Electronic Information, Huazhong University of Science and Technology, Wuhan, 430074 China; 2 Optics Valley Laboratory, Wuhan, 430074 China; 3 School of Precision Instrument and Optoelectronic Engineering, Tianjin University, Tianjin, 300072 China; 4 Key Laboratory of Optoelectronic Information Science and Technology, Ministry of Education, Tianjin, 300072 China

**Keywords:** Contactless integrated photonic probes, Photonic integrated circuits, Silicon photonics, Optical monitoring, Feedback control

## Abstract

On-chip optical power monitors are indispensable for functional implementation and stabilization of large-scale and complex photonic integrated circuits (PICs). Traditional on-chip optical monitoring is implemented by tapping a small portion of optical power from the waveguide, which leads to significant loss. Due to its advantages like non-invasive nature, miniaturization, and complementary metal-oxide-semiconductor (CMOS) process compatibility, a transparent monitor named the contactless integrated photonic probe (CLIPP), has been attracting great attention in recent years. The CLIPP indirectly monitors the optical power in the waveguide by detecting the conductance variation of the local optical waveguide caused by the surface state absorption (SSA) effect. In this review, we first introduce the fundamentals of the CLIPP including the concept, the equivalent electric model and the impedance read-out method, and then summarize some characteristics of the CLIPP. Finally, the functional applications of the CLIPP on the identification and feedback control of optical signal are discussed, followed by a brief outlook on the prospects of the CLIPP.

**Graphical Abstract:**

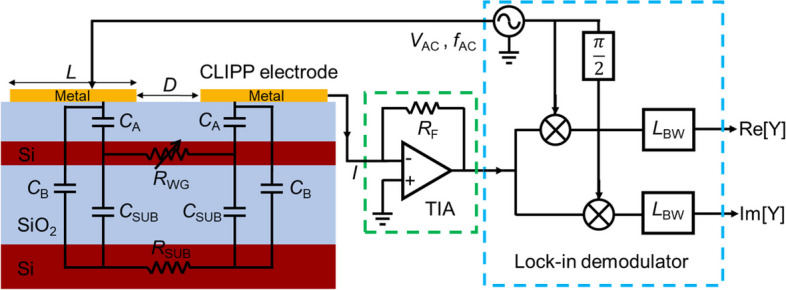

## Introduction

Integrated photonics has been imposing as a key technology in a broad variety of fields of application, such as optical interconnects [1], optical computing [2], bio-sensing [3], and quantum photonics [4]. To achieve various sophisticated optical functionalities, integrated photonics has moved from the single-device level toward large-scale integrated, and complex systems on a chip. Benefiting from its high integration, large bandwidth, low energy consumption, and complementary metal-oxide-semiconductor (CMOS) process compatibility, silicon photonics is mature enough to squeeze several thousands of elements on a single chip [5]. However, the realization of large-scale integration circuits performing complex functions is still a challenge. In fact, a necessary condition for the normal operation of large-scale photonic integrated circuits (PICs) is to monitor, control and stabilize components, which ensures that each component is at the required working point for configuration and is not affected by fabrication tolerances, environmental fluctuations, and mutual crosstalk effects [6], etc. Traditional on-chip optical monitoring is achieved by extracting an additional small portion of optical power from the waveguide and detection by on-chip or external photodetectors [7]. But when thousands of components are integrated into the chip, multi-point light tapping leads to significant loss of output optical power and even affect the functionality of the entire circuit [8]. Therefore, non-invasive optical power monitoring has become particularly important in complex and large-scale PICs.

A non-invasive optical power monitor developed in recent years is the contactless integrated photonic probe (CLIPP) [9], which senses light in the silicon waveguide just by exploiting their natural losses. Compared to other *in situ* optical power monitors, the CLIPP avoids the direct physical contact of the waveguide core through heavily doped regions or electric lines to sweep out carriers from the absorbing region. The CLIPP measures optical power by obtaining the electrical resistance of the core simply though a capacitive access to the waveguide. Currently, the CLIPP has been demonstrated in various PICs to achieve complex functions. In this paper, we present a review of the current development of the CLIPP. We first present the fundamentals of the CLIPP including the equivalent electric model and the read-out method in Section 2. Furthermore, we summarize some characteristics of the CLIPP in Section 3. After that, we discuss the functional applications of the CLIPP in Section 4, and finally conclude this review with a short conclusion and personal outlook on the future development trends of the CLIPP (Sections 5 and 6).

## Fundamentals of the CLIPP

### Concept and principle

**Fig. 1:**
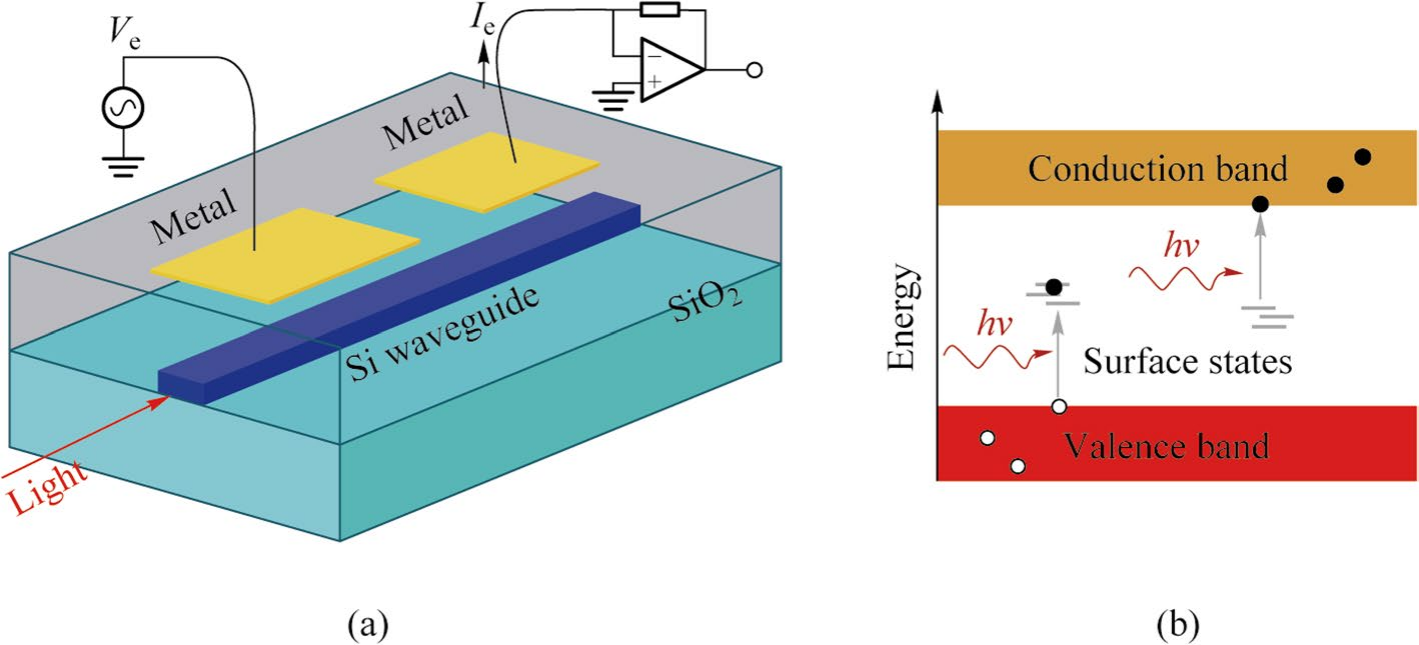
**a** Illustration of the CLIPP device consisting of two metal electrodes deposited onto the upper cladding. **b** Schematic diagram of the mechanism of SSA effect

In 2014, Morichetti et al. first proposed the concept of the CLIPP [9]. Figure 1a shows the schematic of a CLIPP integrated with a channel Si waveguide buried in a silica cladding. The CLIPP consists of two electrodes placed on top of the upper cladding. The simple and non-invasive structure of the CLIPP benefits from the surface state absorption (SSA) effect between the Si waveguide core and SiO_2_ interface. Although material absorption is inhibited when light at wavelengths above 1.1 μm propagates in silicon waveguides, additional free carriers are generated by SSA effect (Fig. 1b). This will cause a change in the local conductivity of the Si waveguide. The higher the local optical power, the more free carriers generated by the SSA effect, and the higher the local conductivity of the Si waveguide, resulting in a higher electric conductance of the local Si waveguide. Therefore, the CLIPP indirectly monitors the optical power in the waveguide by monitoring the conductance variation Δ*G* of the local optical waveguide. In addition, researchers also realized the CLIPP on indium phosphide-based devices by an innovative vertical scheme [10].

To implement a truly non-invasive optical monitoring, the CLIPP exploits a capacitive access to the waveguide, which can obtain the conductance of the local optical waveguide avoiding direct contact with the waveguide or introducing additional doping process.

### Equivalent electric model

**Fig. 2:**
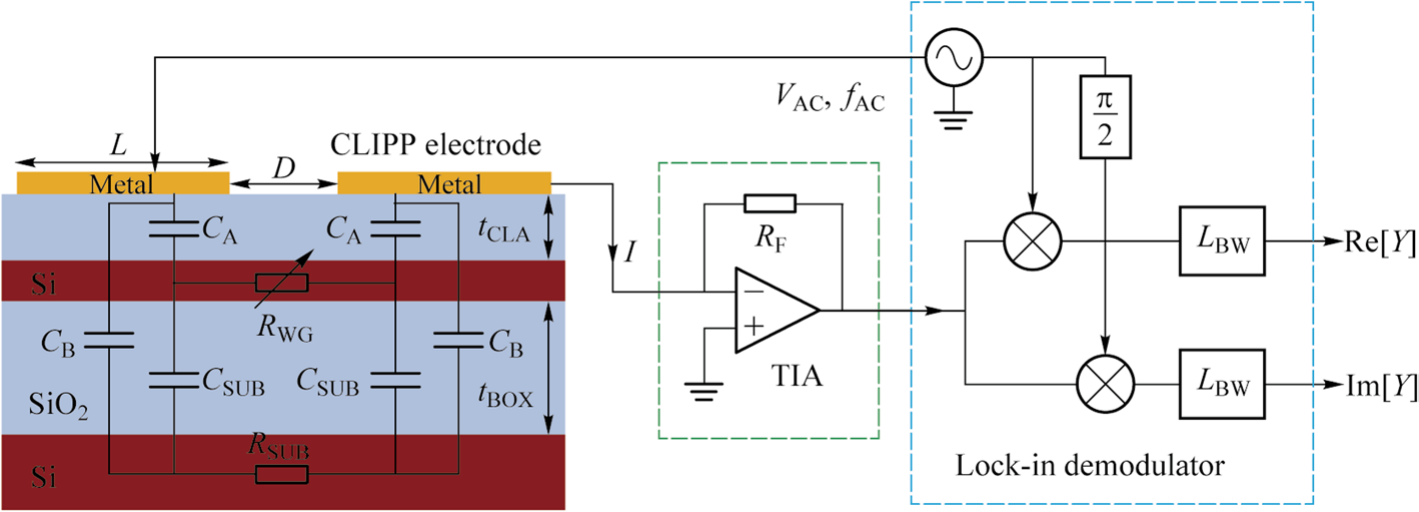
Longitudinal profile of the Si waveguide including the CLIPP equivalent electric model and the impedance read-out system, consisting of a low-noise TIA with feedback resistance *R*_F_ and a lock-in demodulator with bandwidth *L*_BW_

Due to the capacitive access to the waveguide, AC electrical probing is necessary to obtain the conductance of the local waveguide. Therefore, discussing the AC equivalent circuit model of the CLIPP is particularly important for optimization of geometry of the CLIPP and readout of the conductance. Carminati et al. discussed in details the equivalent electrical model of the CLIPP [11]. Figure 2 show the main design parameters and the equivalent electric model of a CLIPP integrated on a Si waveguide buried in a silica cladding. The upper and lower cladding have thickness *t*_CLA_ and *t*_BOX_, respectively. The two same electrodes of the CLIPP have a rectangular shape, with a width *W* and a length *L*, and are separated by a distance *D*. When driving the CLIPP with AC signals, there are three main current paths, the waveguide (*C*_A_-*R*_WG_-*C*_A_), the substrate (*C*_B_-*R*_SUB_-*C*_B_) and the stray capacitance (*C*_E_). *C*_A_, *C*_B_, *R*_WG_, and *R*_SUB_ are access capacitance from the electrodes to the waveguide, capacitance between each electrode and the Si substrate, resistance of the waveguide and resistance of the Si substrate, respectively. *C*_E_ is the parasitic capacitance between the CLIPP electrodes (comprising external bonding and connections). Reference [11] provided the relations between the electrical and geometric parameters of the CLIPP.

When design CLIPPs, the current through *R*_WG_ (modified by the local optical power) with respect to the current through all other electrical elements (not modified by the optical power) should be maximized so that the impedance obtained through AC driving can be approximated as the resistance of the waveguide *R*_WG_. More specifically, the overall impedance of the other two parallel paths needs to be maximized, and the impedance contributed by the series capacitance *C*_A_ on the waveguide path needs to be minimized.

### Impedance read-out

To obtain the conductance of the waveguide, an impedance read-out system is set up in Ref. [9]. As shown in Fig. 2, an AC sinusoidal voltage *V*_AC_ at frequency *f*_AC_ is applied at one electrode of the CLIPP, and the current *I*_AC_ from the other electrode is collected by a transimpedance amplifier (TIA), feeding a lock-in demodulator for the measurement of the complex impedance between the two metallic electrodes. Except for the use of discrete instruments, a low-noise ASIC custom-designed to read 32 CLIPPs was implemented in standard CMOS technology [12].

The selection of driving frequency is crucial, which affects the performance of the CLIPP. Due to the fact that the impedance from the capacitance in the equivalent circuit is related to the driving frequency and the value of capacitance is related to the geometric size of the CLIPP, driving frequency depends on the geometric size of CLIPP.

**Fig. 3:**
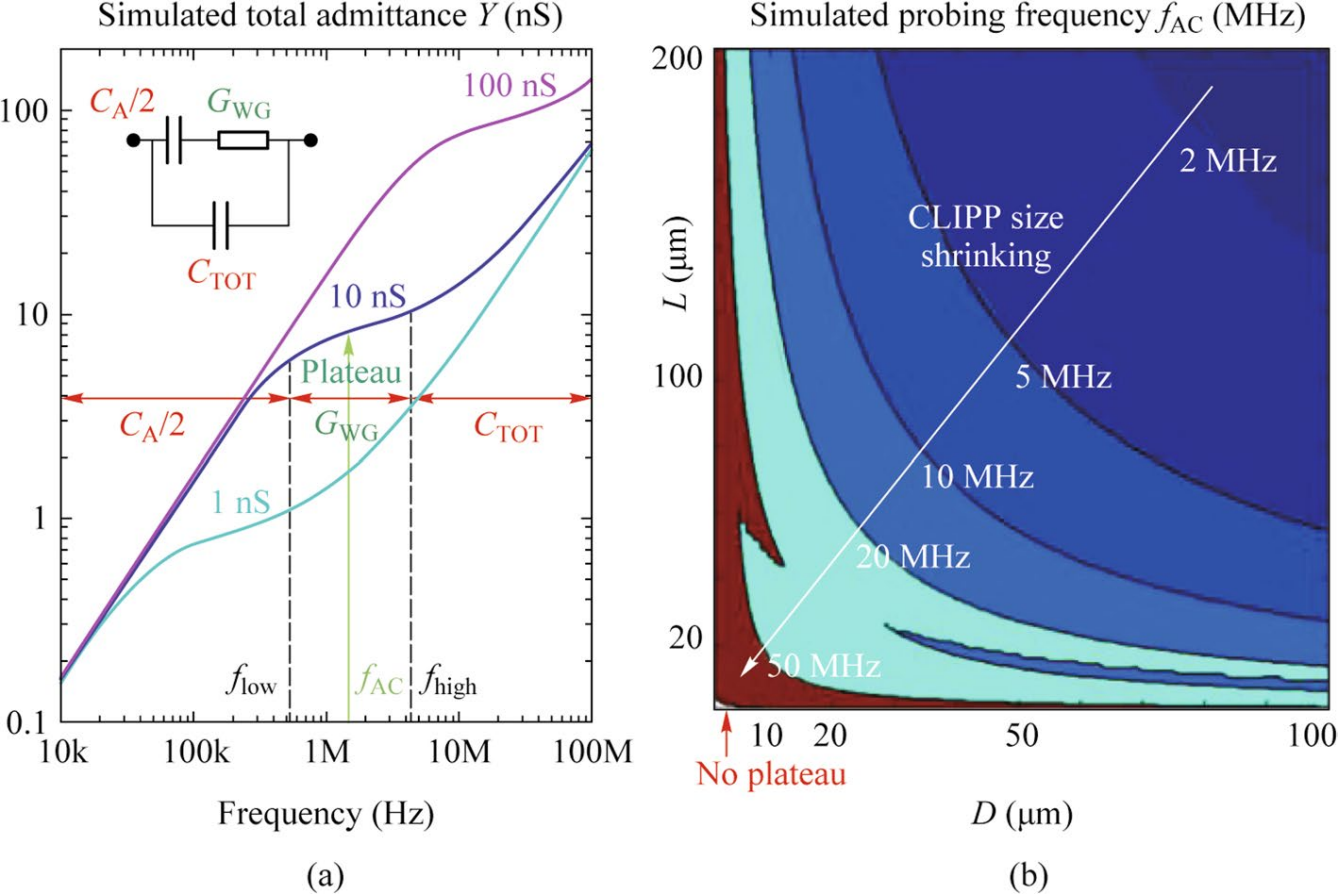
**a** Simulated CLIPP admittance *Y* versus frequency approximated with the lumped model [11]. **b** Relationship between CLIPP electrode size and probing frequency [11]

Reference [11] also provided a range of optimal driving frequencies through simulation and verified it through experiments. There is a plateau (Fig. 3a) that maximizes the current passing through the waveguide path (*C*_A_-*R*_WG_-*C*_A_), and the obtained admittance is related to the optical power inside the waveguide. The lower bound of the plateau is given by *f*_low_ = 1/(2π*R*_WG_*C*_A_/2) ~ 1/*DL* and the CLIPP probing frequency *f*_AC_ should be higher than *f*_low_ to reduce the impedance contributed by the series capacitance *C*_A._ The authors also simulated the interplay between the CLIPP footprint (*L*, *D*) and the corresponding probing frequency *f*_AC_, as shown in Fig. 3b. As we can see, when reducing the size of the CLIPP, the probing frequency *f*_AC_ will increase.

## Characteristics of the CLIPP

### Conductance variation versus optical power

**Fig. 4:**
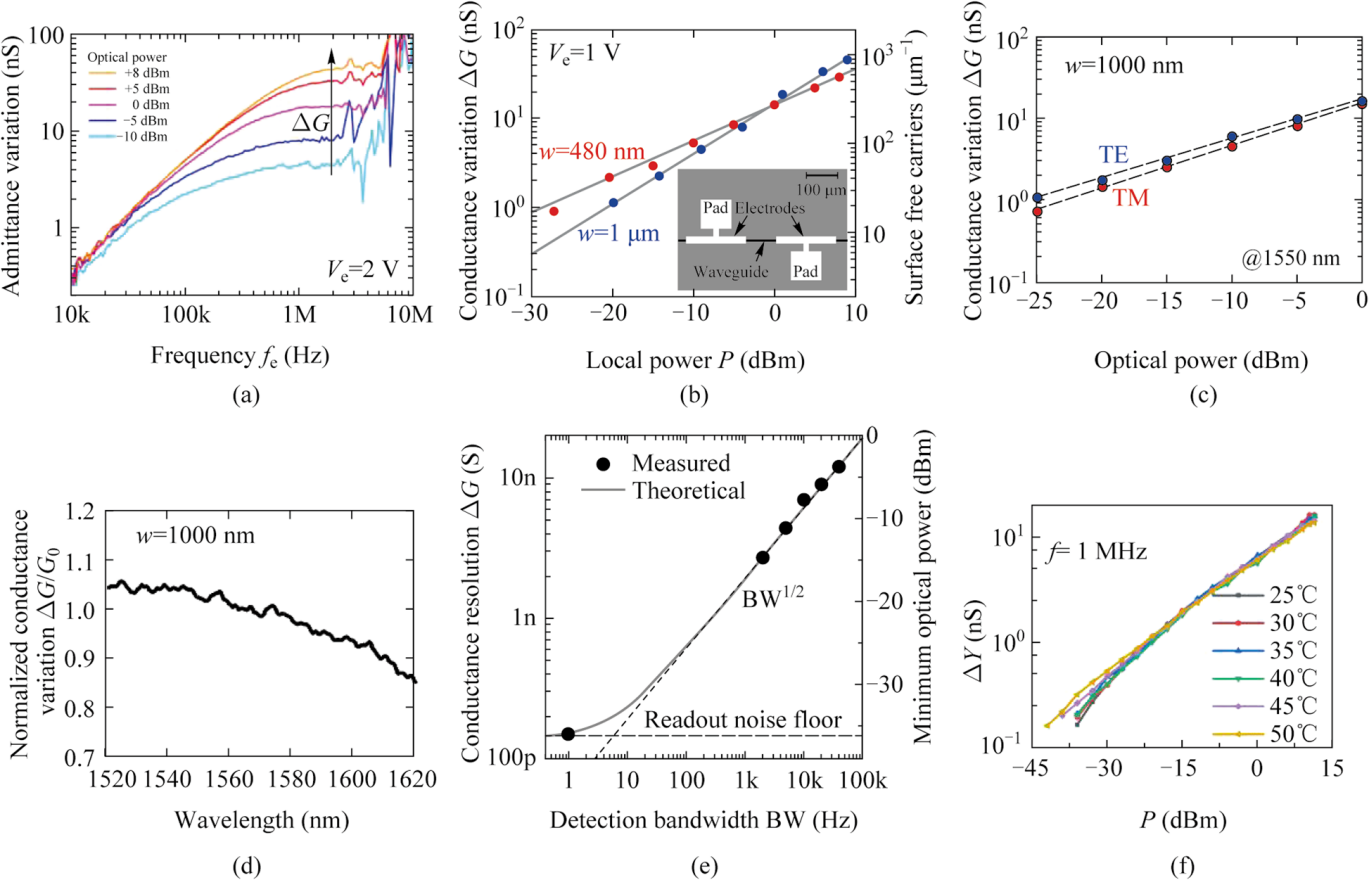
**a** Admittance variation versus the probing frequency under conditions of different optical power [9]. **b** Conductance variation Δ*G* versus the optical power *P* for a single mode and a multimode silicon waveguide [9]. **c** Conductance variation Δ*G* versus the optical power *P* for TE mode and TM mode [13]. **d** Normalized conductance variation Δ*G* as a function of wavelength [13]. **e** Conductance resolution of the CLIPP versus the detection bandwidth of lock-in demodulator [11]. **f** Admittance variation Δ*Y* versus optical power *P* at various temperatures [14]

Morichetti et al. first experiment proved the sublinear relationship between the conductance variation Δ*G* and optical power *P* [9]. They designed and fabricated the CLIPP with metal electrodes (20 μm *×* 200 μm) placed at distance *D* = 100 μm on a single mode (*w* = 480 nm) and a multimode (*w* = 1 μm) silicon waveguide. They measured the admittance variation versus the probing frequency under conditions of different optical power in the waveguide (Fig. 4a) and drove the CLIPPs at *V*_AC_ = 1 V and *f*_AC_ = 1 MHz and conductance variation Δ*G* versus the optical power *P* for the single mode and multimode silicon waveguide was shown in Fig. 4b.

### Low additional loss

Due to the fact that the CLIPP only utilize the natural light loss in the waveguide for monitoring, the additional loss of the CLIPP only comes from metal electrodes induced loss. The relationship between metal induced losses and cladding thickness *t*_CLA_ was simulated in Ref. [11]. The metal induced loss is much lower than the intrinsic propagation loss when the cladding thickness is greater than 700 nm. Obviously, the additional loss of CLIPPs can be basically negligible under the existing CMOS fabrication process.

### Dependence on waveguide geometry

The sensitivity of the CLIPP is related to the density of free carriers generated by SSA per unit length assuming that the photogeneration is homogeneous along the waveguide. Due to the fact that surface states only exist on the waveguide boundaries (Si-SiO_2_ interface), typically within the first three/four Si atomic layers [15], the overlapping integration of waveguide modes and waveguide boundaries is proportional to the number of photogenerated carriers. As shown in Fig. 4b, for both waveguides with *w* = 480 nm (single-mode) and *w* = 1 µm (multimode), the overlapping integration of waveguide modes and waveguide boundaries is similar, which means the total number of photogenerated carriers is comparable. But the high photogenerated carrier density of single-mode waveguides leads to a slightly larger curve slope [13]. Thus, we can increase the overlapping integration and improve the CLIPP sensitivity by appropriately designing the geometry of the waveguide cross-section.

### Polarization dependence

Similarly, for rectangular waveguides with the same width, there is also a slight difference in the overlapping integration of TE polarization and TM polarization with the waveguide boundary. Figure 4c measured admittance variation Δ*G* versus the optical power *P* in a waveguide with width *w* = 1 μm on both TE polarization and TM polarization [13]. The CLIPP exhibits a slightly higher sensitivity on TE polarization than on TM polarization, as shown.

### Wavelength dependence

In Ref. [13], the CLIPP was experimentally demonstrated to be a broadband optical monitor in the 1520−1620 nm wavelength range. Annoni et al. controlled the optical power inside the waveguide to −10 dBm unchanged, then changed the wavelength of the light and measured the relationship between the conductance variation and the wavelength. It was demonstrated that the responsivity of the CLIPP slightly decreases with increasing wavelength within the 100 nm range (Fig. 4d), but the overall performance is comparable.

### Response time and sensitivity

In Ref. [11], researchers pointed out that the response time of CLIPPs is determined by detection bandwidth, the bandwidth of the low-pass filter of the lock-in amplifier. The response time of CLIPP decreases as the detection bandwidth increases. However, an increase in detection bandwidth will result in retaining more noise components during the demodulation, which leads to a decrease in the sensitivity of CLIPP (the minimum detectable optical power). Figure 4e showed the measured minimum detectable conductance variation (calculated as 6 times the root mean square (RMS) value of the measured noise) as a function of demodulation bandwidth. When the detection bandwidth is set to the minimum at 1 Hz, there is a sensitivity limit, which is determined by the inherent noise of the read-out system, especially the input noise from the TIA. Therefore, custom low noise integrated transimpedance amplifiers have been developed to improve the ultimate sensitivity of the CLIPP [12]. Later, Wang et al. conducted experiments to investigate the influence of various parameters in the read-out system on the sensitivity of the CLIPP, including the gain of the TIA and the amplitude and frequency of the probing voltage [16]. The results showed that the sensitivity of CLIPP increases with the increase of the amplitude of the probing signal, the decrease of the probing frequency, and the increase of the gain of the TIA. In addition, the sensitivity of the CLIPP can also be achieved by optimizing the device structure. Grimaldi et al. proposed the square CLIPP employing rib waveguides to reduce the dimension, reaching in this way a higher sensitivity [17].

### Temperature dependence

In Ref. [14], Zhang et al. characterized the CLIPP at various temperatures, ranging from 25^◦^C to 50^◦^C. The results indicated that in the absence of light, the measured admittance value slightly increases with increasing temperature. Under the condition of light transmission, admittance variation Δ*Y*, as a function of optical power, *P*, at various temperatures showed only small differences (Fig. 4f). This indicated that the performance of the CLIPP is not very sensitive to temperature changes ranging from 25^◦^C to 50^◦^C in the wavelength range of 1550 nm.

## Functional applications of the CLIPP

The CLIPP is widely used in photonic integrated circuits due to its advantages such as simple structure, small size, and non-invasiveness, and has shown the potential to expand into free-space optics. Table 1 summarizes the main applications of CLIPP in various fields in recent years. This section will focus on the applications of the CLIPP in fiber-to-waveguide alignment, on-chip optical signal identification and manipulation, dithering‐based real‐time feedback control of photonic devices and extension to free-space optics.

**Table 1:** Summary of the main applications of CLIPPs

Related technologies	Functional applications	References
Custom closed-loop algorithm	Fiber-to-waveguide alignment	[18]
Pilot tones	Monitoring of mode-multiplexed channels	[19]
Pilot tones	Tuning of micro-ring filter array for reconfigurable add-drop	[20]
Pilot tones	Light-path tracking and routing	[21]
Pilot tones	Unscrambling of optical modes	[22]
Pilot tones	OSNR monitoring	[23]
Dithering	Wavelength locking of micro-ring resonators	[24]
**–**	Wavelength locking of a WDM transmitter	[25]
Dithering	Wavelength locking of cascaded double-ring resonators	[26]
Dithering	Working point locking of micro-ring modulators	[27]
Dithering	Wavelength locking of micro-ring resonators and working-point locking of micro-ring modulators	[28]
Normal-incidence	Four quadrant photodetector	[29]

### Fiber-to-waveguide alignment

Simple, efficient and accurate fiber-to-waveguide alignment has always been a crucial aspect for the testing and packaging of PICs, which impacts on the performance, cost, reliability and manufacturability of the photonic system [30, 31].

The traditional active alignment process requires to have two optical fibers simultaneously aligned to the integrated waveguide, one on the side of the laser, the other on the side of the photodetector. Only when the positions of both fibers are optimized can the photodetector find the maximum coupling optical power. Meanwhile, the complexity of the optical circuit will lower the optical power received. These undoubtedly increase the difficulty of the alignment process.

**Fig. 5:**
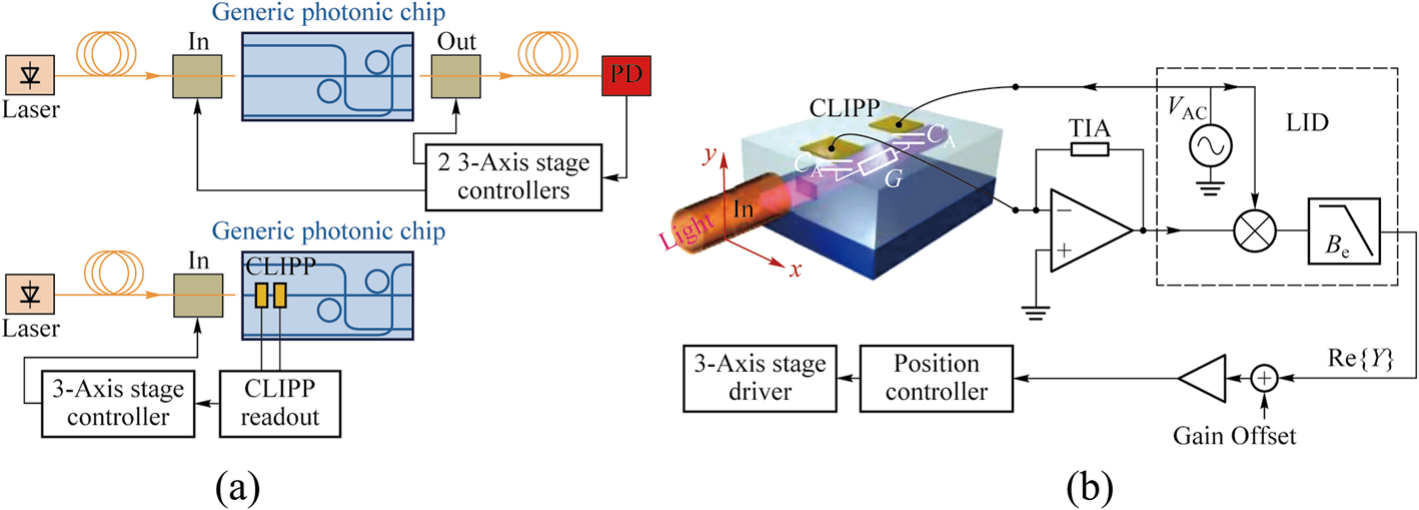
**a** Conventional approach and the original active technique assisted by the CLIPP for the alignment to photonic chips [18]. **b** Feedback system using the CLIPP monitoring signal to control the fiber-to-waveguide alignment position [18]

Carminati et al. presented an original active technique for single fiber alignment exploiting the CLIPP [18]. As shown in Fig. 5, the CLIPPs were placed in proximity of the input facet to make the alignment process independent of the optical circuit and to eliminate the need for the fiber and the photodetector at the output end. Accurate (40 nm) and fast (few seconds) automated fiber alignment was shown on a silicon photonic chip with a custom closed-loop algorithm. Additionally, the technique could apply both to edge and vertical coupling to other semiconductor optical waveguides [18].

### On-chip optical signal identification and manipulation

On-chip optical power monitoring is essential for the control and stabilization of the photonic system such as routing and switching of optical channels [32] and self-configuring of PICs [33]. However, in practical applications, most of these functions require identifying the required optical signal from multiple channels or optical noise, and then, monitoring and manipulating it.

The technology of pilot tones based on the CLIPP can achieve the identification of specific optical signals while ensuring the non-invasive nature of the entire process. To this aim, at the transmitter end, the required optical signal is labeled by introducing a small modulation of intensity. The tone should be a small percentage (modulation depth) of the signal amplitude to avoid affecting the performance of transmitted signals, and be at a frequency below the driving frequency of the CLIPP, about few kHz, not interfering with the GHz digital modulation of telecommunication systems. The CLIPP output electric signal needs to be double demodulated: first at the driving frequency of the CLIPP and then at the frequency of the tone, as shown in Fig. 7c [23]. After each of mixers, there is a low-pass filter. The bandwidth of the first filter must allow the components of frequency of tone to pass. The second filter needs to allow the DC component to pass so the bandwidth can be set as narrow as possible to obtain higher sensitivity at the cost of response time of the CLIPP. In this way, the voltage finally demodulated is only related to the optical power of the required signal.

**Fig. 6:**
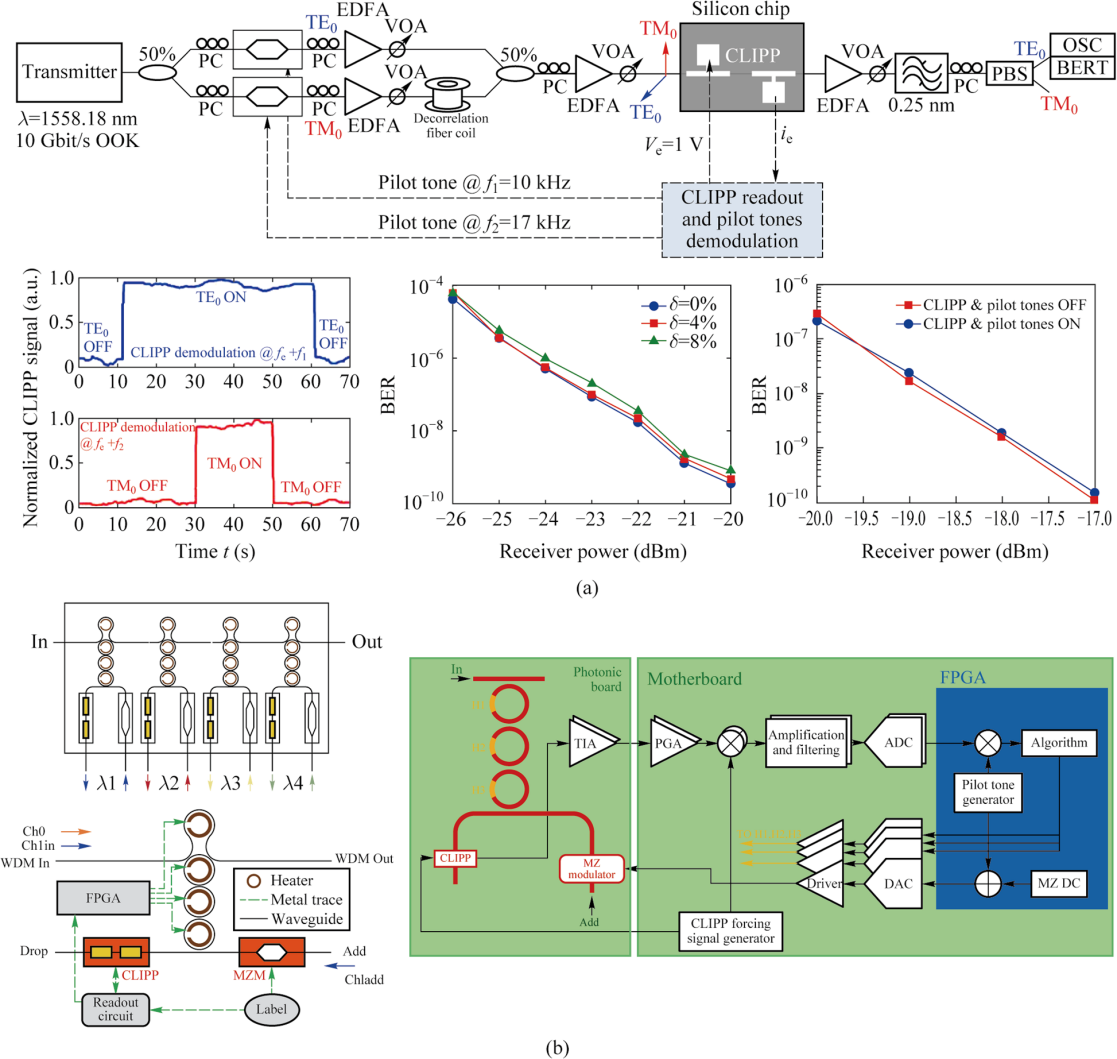
**a** Simultaneous CLIPP monitoring and identification of two mode-multiplexed channels labeled by different pilot tones and negligible impact on the communication system caused by the CLIPP and pilot tones [19]. **b** Scheme of 4 channel micro-ring hitless based filter array and the feedback system for the add-drop of a data transmitting channel [20]

In Ref. [24], this technology has been demonstrated for the first time to swap the resonator wavelength between two optical signals at wavelengths *λ*_1_ and *λ*_2_ injected in the micro-ring. Not long after, Grillanda et al. applied this technology to non-invasive on-chip monitoring of mode-division-multiplexing (MDM) channels [19]. They implemented the discrimination and the simultaneous monitoring of two channels transmitted at the same wavelength and multiplexed on the fundamental transverse electric and magnetic modes of the silicon waveguide by labeling each signal respectively. Neither the modulation of the pilot tone nor the driving of the CLIPP introduces any penalty to the system performance (Fig. 6a). And the work in Ref. [20] showed the reconfigurable add-drop of a data transmitting channel labeled by tone in a silicon micro-ring filter array without affecting other channels. This technology can be extended to more complex multi dimensions of light field multiplexing/demultiplexing system.

**Fig. 7:**
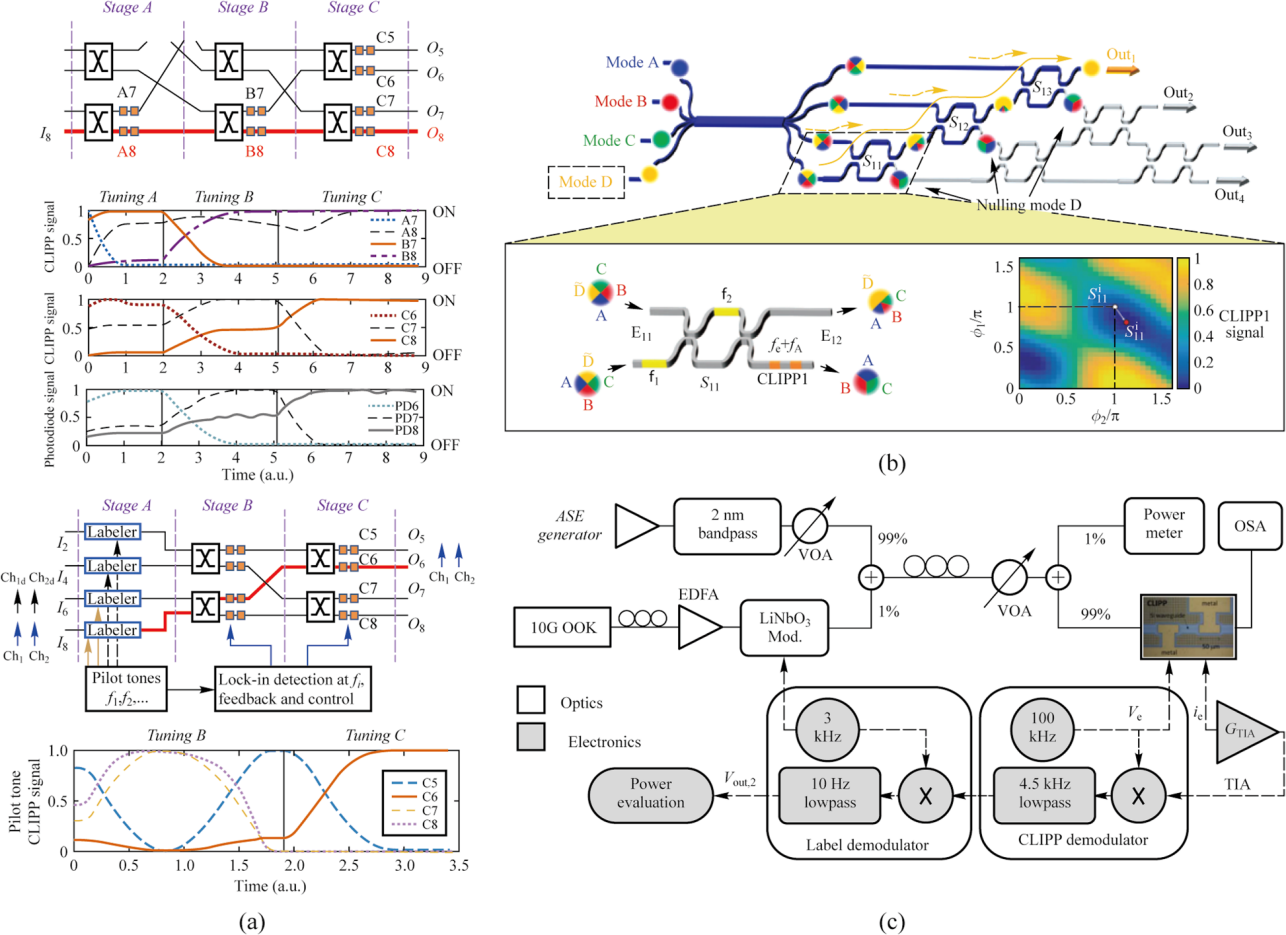
**a** CLIPP-assisted light-path tracking and routing of concurrent signals discriminated by on-chip labeling with pilot tones [21]. **b** On-chip unscrambling of optical modes labeled with pilot tones assisted by CLIPPs [22]. **c** Setup diagram of on-chip OSNR monitoring with the CLIPP and the two-step lock-in demodulation read-out method of signal labeled [23]

Researchers also used CLIPPs to monitor the state of each switching element individually in real time in an 8 × 8 silicon photonic switch fabric based on Mach–Zehnder interferometers (MZIs) [21]. Light-path tracking and circuit reconfiguration were demonstrated by sequential tuning and feedback control assisted by a multichannel integrated ASIC [12] for CLIPPs read-out (Fig. 7a). In addition, the routing was also implemented with several input signals simultaneously present by the pilot tone method based on the CLIPP. Similarly, on-chip unscrambling of MDM channels was demonstrated in a 4 × 4 triangular array or mesh of 6 tunable 2 × 2 beam splitters and 5 CLIPPs [22]. The modes were labeled by different tones respectively and the CLIPPs were double demodulated to discriminate these modes and tune the MZI beam splitters (Fig. 7b). Another important application scenario was on-chip the in-band optical signal to noise ratio (OSNR) monitoring of a transmission channel [23]. OSNR measurement from 8 up to 27 dB/0.1 nm on 10-Gb/s ON–OFF keying signals was demonstrated, which is equivalent to the measurement from an external optical spectrum analyzer.

### Dithering-based real-time feedback control of photonic devices

Due to fabrication tolerances, environmental fluctuations, and mutual crosstalk effects, self-configuration with a closed-loop feedback control of single or cascaded devices in large-scale PICs are necessary prerequisites for achieving various complex and reconfigurable functions. The CLIPPs combined with the dithering technology were found to be suitable for complex photonic architectures consisted of cascaded devices, without any impact of crosstalk and any calibration [34].

**Fig. 8:**
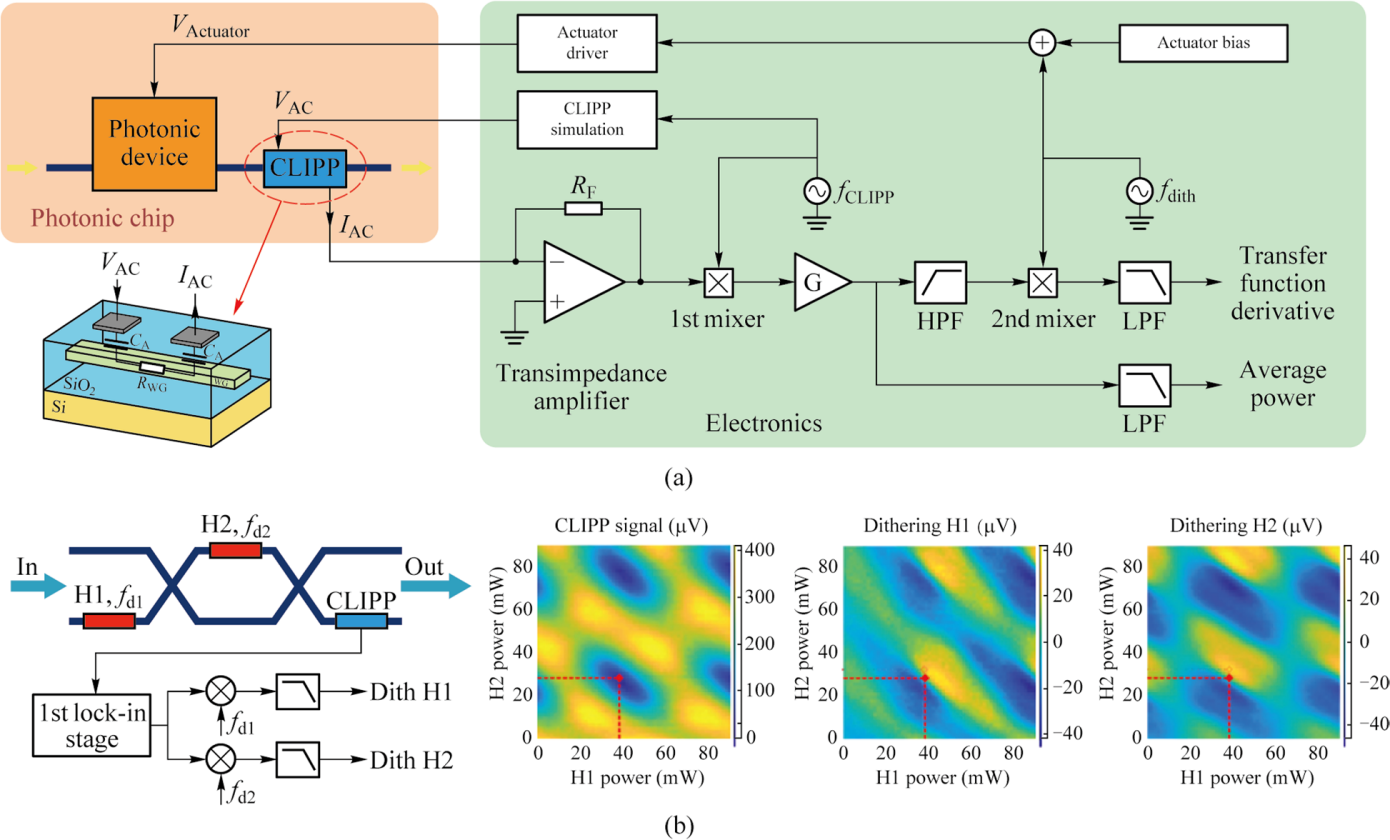
**a** Schematic of two-step lock-in demodulation read-out method to extract the derivative of the transfer function at the working point *V*_ACTUATOR_ with the CLIPP [34]. **b** Orthogonal modulations added to every heaters on the MZI and separate demodulation in the second stage to obtain the partial derivatives of the transfer function [34]

The dithering technology [35] is a method of directly obtaining the derivative of the transfer function of tunable photonic devices, which is achieved by superimposing a sufficiently small dithering signal to the control voltages of the actuators of the photonic device. In this way, the output optical power is also modulated by the frequency of the dithering signal and the amplitude of modulation is directly proportional to the derivative of the transfer function at the working point. When the output is detected by a CLIPP, the two-stage lock-in demodulation mentioned above can also be used to extract the amplitude of frequency component of the dithering signal, as shown in Fig. 8a [34]. In addition, orthogonal modulations and separate demodulation are used to discriminate the effect of different actuators for devices controlled by multiple actuators [34].

**Fig. 9:**
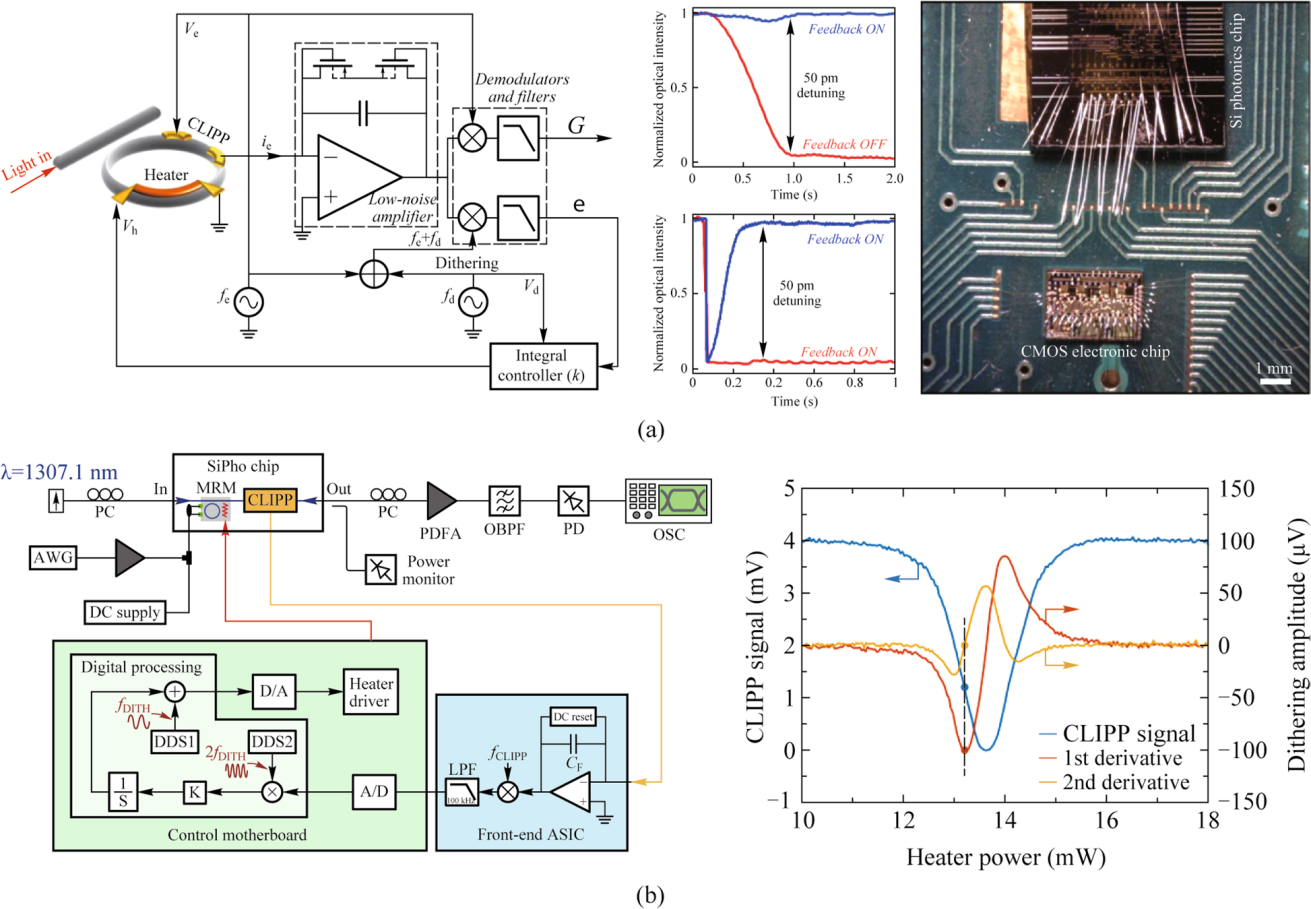
**a** Automatic wavelength locking of the micro-ring resonator and CMOS electronic chip that performs the readout of the CLIPP electric signal [24]. **b** Setup diagram of locking and stabilizing the working point of a silicon micro-ring modulator and transfer function of the micro-ring and its first and second derivatives obtained by the dithering technology [27]

Grillanda et al. first combined CLIPPs with dithering technology and applied to automatic wavelength locking of micro-ring resonators (Fig. 9a) [24]. In contrast, micro-ring wavelength locking platforms with CLIPPs and dithering-free were applied to the feedback control of a wavelength-division-multiplexing (WDM) transmitter based on directly modulated lasers, which have lower locking accuracy [25, 36, 37]. Later, researchers demonstrated wavelength locking of cascaded double-ring resonators in the multiplexer and demultiplexer to counteract temperature and wavelength drift in a dual-socket interconnect system based on the arrayed waveguide grating router (AWGR) [26, 38].

As an extension of dithering technology, Grimaldi et al. proposed a power-independent and calibration-free solution to stabilize the working point of a silicon micro-ring modulator [27]. They locked the working point where the derivative of the transfer function is maximum by extracting the second-derivative signal and setting it to zero through the feedback loop with the CLIPP monitoring, as shown in Fig. 9b. The second-derivative signal extracted by demodulating the signal at twice the dithering frequency at the second lock-in stage, resulting from the intrinsic nonlinearities of photonic devices. The work in Refs. [28, 39] implemented the wavelength locking of micro-ring resonators and working-point locking of micro-ring modulators simultaneously in AWGR-based interconnect system equipped with transmitters and validated the system robustness.

Except for micro-ring-based circuits, dithering technology with CLIPPs was also used to self-configurate MZI-based photonic processors automatically and the reconfiguration time of each MZI is about 5 ms [40, 41].

### Extension to free-space optics

**Fig. 10:**
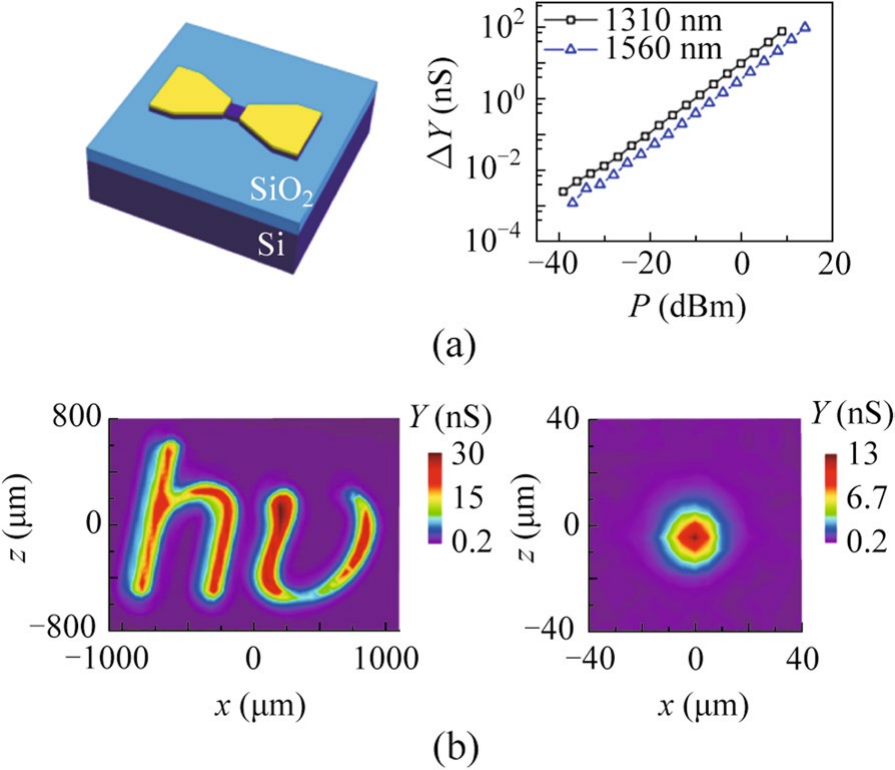
**a** Schematic diagram of the normal-incidence infrared photoconductor based on the SSA effect and the Conductance variation Δ*G* versus the optical power *P* [42]. **b** Applications of normal-incidence infrared photoconductor in infrared imaging and beam-profile measurement [42]

In recent years, researchers have extended the concept of the CLIPP to free-space optics [14, 29, 42, 43]. In 2021, Wang et al. proposed a normal-incidence infrared photoconductor based on the SSA effect in silicon [42]. They made a photoconductor with a photosensitive area of 5 µm × 5 µm and measured admittance variation as a function of incident optical power at wavelengths of 1560 and 1310 nm (Fig. 10a). The result showed a response similar to the CLIPP. They also demonstrated its applications in infrared imaging and beam-profile measurement (Fig. 10b). The photoconductor was expected to expand to multi-pixel arrays or transparent infrared cameras with high sensitivity of −46 dBm. Later, they fabricated a silicon four quadrant photodetector operating at wavelength of 1550 nm and applied it to the measurement of beam position and deflection angle [29]. Experiment proved that the device can also operate at the shortwave of 780 nm.

## Future perspectives

**Fig. 11:**
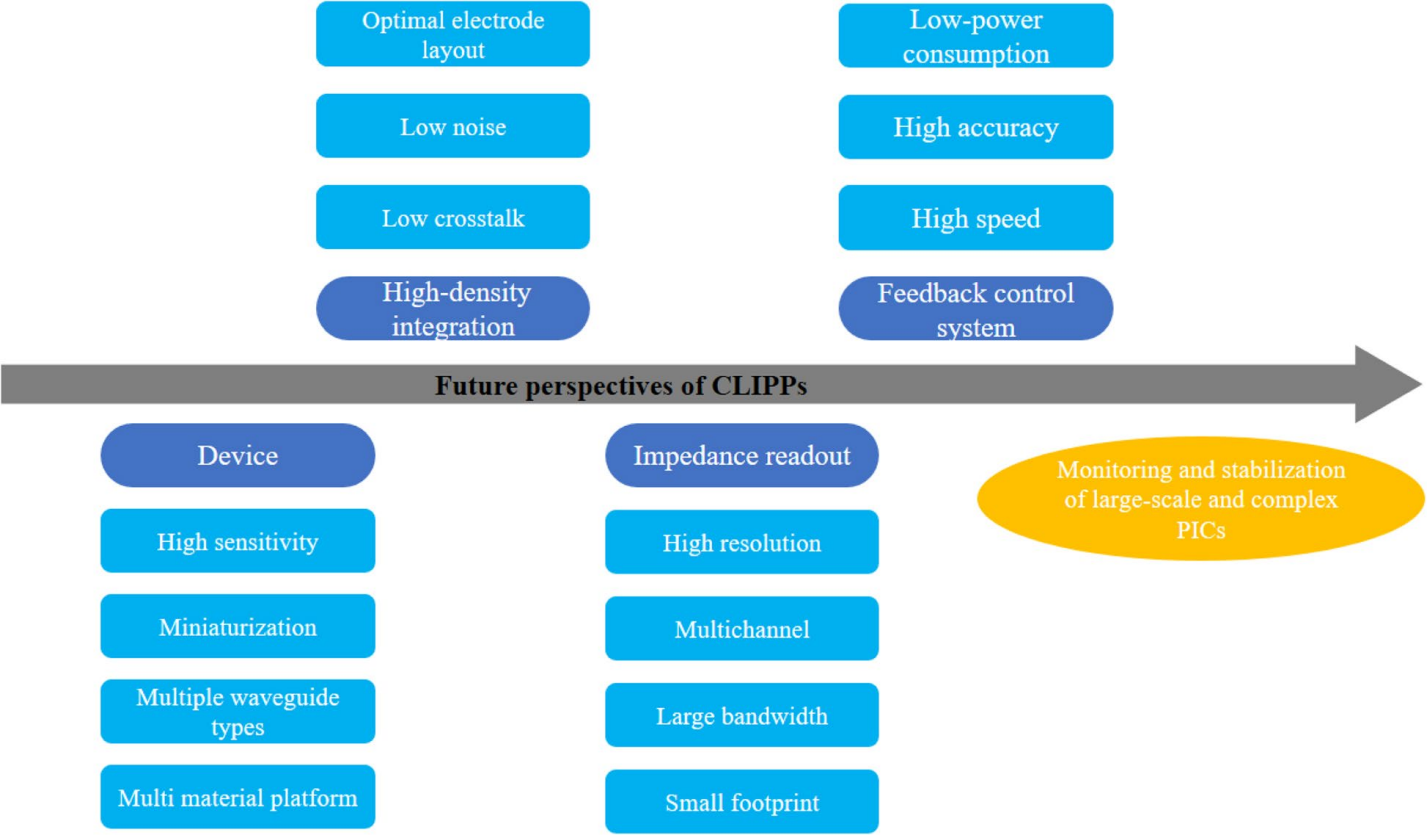
Future development directions of CLIPPs

As a non-invasive optical power monitor, the CLIPP has significant application prospects in large scale complex PICs. However, there are still some steps left before CLIPPs can be applied on a large scale to any PICs. The future development directions of the CLIPPs are shown in Fig. 11.

Firstly, the development directions of device level the CLIPPs mainly revolve around four directions: high sensitivity, miniaturization, multi waveguide types, and multi material platforms. High sensitivity and miniaturization are essential for optical signals monitoring in large-scale PICs, usually by optimizing the structure of the device to increasing the access capacitance can shorten the waveguide length to achieve miniaturization and high sensitivity of CLIPPs simultaneously. Additionally, CLIPPs on multi waveguide types and multi material platforms are worthy of further study. They are also the foundation for CLIPPs to be competent in various PICs.

Secondly, the high-density integration of CLIPPs is an inevitable trend for large-scale PICs. Due to the presence of the substrate, the light coupled to the photonic chip will flood the substrate which will be detected by other CLIPPs in the circuit and cause crosstalk. Reducing crosstalk between CLIPPs is currently a problem that needs to be addressed. The routing from the CLIPP electrodes to the bonding pads should also be optimized to achieve low noise and small footprint for high-density integration of CLIPPs.

Thirdly, the impedance readout system of the CLIPPs largely determines the performance of the CLIPPs, for example, high resolution impedance readout system can improve the sensitivity of CLIPPs, and large bandwidth system can help reduce the size of CLIPPs. And multi-channel small footprint impedance readout system is a prerequisite for the high-density integration of CLIPPs. Therefore, developing impedance readout ASICs with high resolution, multi-channel, large bandwidth, and small footprint is an important challenge.

Last but not the least, the feedback control system based on CLIPPs is the key to maintaining device stability in large-scale PICs. The new feedback system should be designed with faster response speed to resist the faster environmental fluctuations without sacrificing control accuracy. In addition, the reduction of power consumption in feedback systems based on CLIPPs in high-density integration is also a challenge. Integrating the entire control system into an ASIC is a highly promising direction for achieving low power consumption, without using any controllers outside of the chips. Most significantly, the monolithic photonics-electronics integration of complex reconfigurable PICs equipped with CLIPPs is a new exciting frontier of innovation.

## Conclusions

The CLIPPs have been widely used in large-scale PICs in recent years. In this review, we summarized the fundamentals of the CLIPP, the characteristic performance of the CLIPP and its applications combined with pilot tone and dithering technology on the recognition, monitoring, and feedback control of on-chip optical signals. Finally, we discussed several challenges remaining for large-scale practical applications of the CLIPPs. We believe that the future development of CLIPPs may head to four directions: device, high-density integration, impedance readout, feedback control system.
